# Psychometric Properties of the Metacognitions About Online Gaming Scale in the Chinese Population and Its Relationship With Internet Gaming Disorder: Cross-Sectional Study

**DOI:** 10.2196/45985

**Published:** 2024-04-22

**Authors:** Shuhong Lin, Xinxin Chen, Linxiang Tan, Zhenjiang Liao, Yifan Li, Ying Tang, Qiuping Huang, Hongxian Shen

**Affiliations:** 1 Department of Psychiatry Second Xiangya Hospital of Central South University Changsha China; 2 National Clinical Research Center for Mental Disorders Changsha China; 3 Education Center for Mental Health Central South University Changsha China; 4 School of Humanities and Management Hunan University of Chinese Medicine Changsha China

**Keywords:** metacognition, metacognitions about online gaming, Internet Gaming Disorder, psychometric properties, Chinese

## Abstract

**Background:**

Metacognitions about online gaming have been shown to be correlated with Internet Gaming Disorder (IGD). Knowledge of metacognitions about online gaming can help to understand IGD. The Metacognitions about Online Gaming Scale (MOGS) is a reliable and valid tool to measure specific metacognitions about online gaming in both adults and adolescents, which is lacking in China.

**Objective:**

This study was conducted to assess the psychometric properties of the Chinese version of the MOGS (C-MOGS) and its relationship with IGD in the Chinese population.

**Methods:**

A total of 772 Chinese individuals (age: mean 21.70, SD 8.81 years; age range: 13-57 years; 458/772, 59.3% male) completed a web-based questionnaire survey, including the C-MOGS and a battery of validated scales measuring IGD, gaming motives, depression, and anxiety.

**Results:**

Through exploratory and confirmatory factor analyses, the 3-factor structure was confirmed to have adequate model fit and internal consistency reliability (Cronbach α≥.799, Guttman split-half coefficients≥0.754). Concurrent validity of the C-MOGS was supported by its correlations with IGD (*P*<.001), gaming motives (*P*<.001), depression (*P*<.001), and anxiety (*P*<.001). Furthermore, the incremental validity analysis showed that the C-MOGS predicted 13% of the variance in IGD while controlling for gender, age, weekly gaming hours, gaming motives, depression, and anxiety.

**Conclusions:**

This study provides evidence that the psychometric properties of the C-MOGS are appropriate and emphasizes its positive association with IGD. The C-MOGS is a reliable and valid instrument for mental health workers to assess metacognitions about online gaming in the Chinese population.

## Introduction

Metacognition refers to the awareness of one’s own thoughts and behaviors, as well as the ability to monitor and alter behavior. It encompasses any cognitive process that receives information from and exerts a controlling influence on another cognitive process [1-4]. More specifically, it comprises metacognitive knowledge and metacognitive regulation. Metacognitive knowledge refers to information and beliefs about one’s cognitive processes, while metacognitive regulation pertains to skills to regulate thoughts, including planning, supervision, and regulation [5]. Metacognition contributes to effective decision-making across a variety of contexts [4]. For instance, it facilitates the smooth operation of ongoing thought and behavior by helping us recognize our errors [6], regulate the deployment of executive function [7], and detect lapses of attention [8]. Originating from cognitive psychology, metacognition has been linked to psychological disturbances [9,10].

In recent years, studies have highlighted the potential role of metacognitions in the development of addictive behaviors, such as problematic gaming behavior [11,12]. However, due to the lack of suitable research instruments, conducting further investigations in China has been challenging. To address this issue, this study aimed to evaluate the validity of the Metacognitions about Online Gaming Scale (MOGS) [13] among the Chinese population and its association with gaming behavior.

According to the self-regulatory executive function model, metacognitions play a critical role in the occurrence and development of psychological dysfunction [14]. In this model, psychological dysfunction is activated and perpetuated by a fixed thinking pattern called cognitive attentional syndrome (CAS), which comprises several maladaptive coping strategies (eg, rumination, threat-monitoring, and avoidance). The CAS is driven and maintained by maladaptive metacognitions [15]. Maladaptive metacognitions mistakenly regard the CAS as an effective coping style, resulting in a vicious cycle of ineffective self-regulation [16]. Over the last 40 years, metacognitions have been associated with several mental and psychological problems [17], such as obsessive-compulsive disorder, schizophrenia, addiction, anxiety, and depression [18-20].

In the domain of addictive behaviors, metacognitions are divided into 2 subtypes: positive and negative [21]. The former refers to the beliefs that engaging in specific addictive behaviors is a strategy of affective and cognitive self-regulation, such as “Drinking helps me think more clearly” and “Gambling can improve my mood” [22,23]. The latter refers to the concerns about the uncontrollability and danger of thoughts or engagement with addictive behaviors. For example, “Drinking will interfere with my thought” and “Once I start thinking about drinking, I cannot stop” [24]. Previous studies have shown that positive metacognitions can motivate addictive behaviors in the early stage, while negative metacognitions contribute to their perpetuation by activating negative emotional states as a reinforcement [11,21]. In recent years, metacognition has been correlated with many addictive behaviors, such as problematic alcohol use [25-27], nicotine dependence [28,29], gambling disorder [30-32], problematic Internet use [33-35], problematic social media use [36-38], and Internet Gaming Disorder (IGD; problematic online gaming) [39,40].

As an addictive behavior, IGD was first included in the research appendix section of the Fifth Edition of the Diagnostic and Statistical Manual of Mental Disorders (DSM-5) in 2013 [41], then it was officially included in the addiction disease unit of the *Eleventh Revision of the International Classification of Diseases (ICD-11)* in 2018 [42]. Its core characteristics include losing control while gaming, prioritizing gaming over other interests, and causing functional damage in daily life. Excessive online gaming results in various problems, such as sacrificing real-life relationships, sleep, work, and education, leading to brain damage [43-46]. According to a recent review, the global prevalence of IGD was 3.05%, and it was higher among Asians (5.08%) than Europeans (2.72%) [47]. In China, the prevalence ranges from 3.5% to 17%, which is higher than the global average level [48-50].

In order to effectively prevent and treat this disorder, extensive research has been conducted to investigate its etiology. These studies have revealed a significant association between IGD and various psychological factors, including negative affect, gaming motives, and maladaptive cognition [51]. The Interaction of Person-Affect-Cognition-Execution (I-PACE) model proposed by Brand et al [52,53] suggests that the initiation of addictive behaviors arise from the integration of emotional and cognitive responses to internal or external stimuli along with specific motivations. Motives are sets of knowledge that represent the emotional preferences expressed in our thoughts and concepts. Gaming motives could be considered as stimulating factors of gaming behavior, which may play an important role in the development of IGD [51]. Furthermore, Spada et al [21] posited that the development and persistence of addictive behaviors, including IGD, are strongly influenced by particular metacognitions about addictive behaviors.

According to previous studies, metacognitions have been associated with IGD [38,39]. However, these studies mainly focused on generic metacognitions (eg, beliefs about worry, cognitive monitoring, the need for thought suppression). To assess specific metacognitions about online gaming, Spada and Caselli [13] developed a self-rating instrument called the Metacognitions about Online Gaming Scale (MOGS). In the original validation of the MOGS, an exploratory factor analysis (EFA) was performed with 225 adults in Study 1 that suggested a 2-factor solution: Negative Metacognitions about Online Gaming (N-MOG; 6 items) and Positive Metacognitions about Online Gaming (P-MOG; 6 items) [13]. The N-MOG assesses negative metacognitions about the uncontrollability and danger of thoughts on gaming. The P-MOG measures positive metacognitions in which online gaming helps individuals regulate affect and thought. In Study 2, the confirmatory factor analysis (CFA) with another sample of 348 individuals further divided N-MOG into 2 factors and built a 3-factor structure: Negative Metacognitions about the Uncontrollability of Online Gaming (N-MOGU), Negative Metacognitions about the Dangers of Online Gaming (N-MOGD), and P-MOG [13]. All 3 factors reported adequate internal reliability (Cronbach α≥.79). While exploring predictive validity, the study showed that MOGS was positively related to gaming hours and Internet addiction [13]. Overall, these findings demonstrated the reliability and validity of the MOGS.

To extend the utility of the MOGS to adolescent populations from other countries, Akbari et al [54] translated it into Persian and evaluated its psychometric properties among 769 Iranian adolescents. The results showed that the 3-factor structure had appropriate construct validity and internal consistency (Cronbach α≥.79). Furthermore, metacognitions about online gaming were able to independently predict problematic gaming behavior while controlling for personality traits, gaming motives, gaming-related cognitions, and negative affect [54]. Another study investigating the association between IGD and social anxiety reported that metacognitions about online gaming were significantly correlated with IGD and mediated the latter’s relationship with social anxiety [55].

These studies indicated an association between specific metacognitions about online gaming and IGD. Further exploration could be beneficial for the treatment and prevention of IGD, especially in countries with a higher prevalence, such as China. However, it is difficult to conduct relevant research in China because of the lack of instruments used to evaluate specific metacognitions. Therefore, the primary objective of this study was to translate the MOGS into Chinese and validate its psychometric properties among Chinese adolescent and adult gamers using online convenience sampling. Additionally, the study aimed to investigate the unique influence of metacognitions about online gaming on IGD while considering variables such as anxiety, depression, and motivation. The hypothesis was that, within the Chinese population, positive and negative metacognitions about online gaming would serve as independent risk factors for IGD, distinct from other contributing factors.

## Methods

### Participants

We recruited all individuals online through convenience sampling in June 2021. The inclusion criteria were as follows: (1) age ≥13 years, (2) Chinese speakers who could understand the questionnaires, (3) consent to participate (adolescents with parental consent), and (4) played games at least one hour every week (excluding online gambling) in the last 12 months.

In total, 996 individuals participated in this survey. We excluded 88 individuals whose answer was “No” to the item “Are your answers to this questionnaire true and reliable?”, 37 who gave the same answers to more than 50% of the questions and whose time spent on the questionnaire was less than the mean minus 3 SD, and 99 who were younger than 13 years old. The final sample included 772 participants.

### Ethical Considerations

Before starting the anonymous online investigation, participants were informed about the purpose and rights of the study and signed an online informed consent form. Those younger than 18 years needed to inform their guardians and obtain consent before filling out the questionnaire. The ethics committee of the Second Xiangya Hospital of Central South University approved this study (protocol code 2020004; dated March 1, 2020).

### Measures

#### Basic Information

Basic information included sociodemographic information and Internet gaming characteristics. The former included gender, age, employment, years of education, and family structure (eg, single-child family). For the latter, participants reported their average time spent gaming (weekly gaming hours), gaming devices (a multiple-choice question), the number of long-term gaming partners, and self-evaluation of gaming addiction.

#### Metacognitions About Online Gaming

Metacognitions about online gaming were measured using the MOGS [13], which contains 12 items rated on a 4-point Likert scale (1=Do not agree to 4=Agree very much). The MOGS comprises the following 3 factors: (1) N-MOGU (3 items, such as “Once I start online gaming I cannot stop”), (2) N-MOGD (3 items, such as “Online gaming makes me lose control”), and (3) P-MOG (6 items, such as “Online gaming stops me from worrying”). A higher score indicates a higher degree of specific metacognition about online gaming.

#### IGD Symptoms

The severity of IGD symptoms was assessed using the Internet Gaming Disorder Scale-Short Form (IGDS9-SF) [56,57]. The IGDS9-SF is a 9-item scale developed from the core symptoms of IGD proposed by the DSM-5 and assesses gaming activities and their adverse effects in the past 12 months. All items are rated on a 5-point Likert scale (1=never to 5=very often). The scores range from 9 to 45. Higher scores represent more severe IGD symptoms. With adequate reliability (Cronbach α≥.9), the Chinese version of the IGDS9-SF was used in our research [58,59]. The Cronbach α was .90 in this study.

#### Gaming Motives

We assessed gaming motives using the Motives for Online Gaming Questionnaire (MOGQ) [60]. It includes 27 items comprising the following 7 motivational dimensions (all rated on a 4-point Likert scale): escape, skill development, recreation, competition, coping, fantasy, and social. The Chinese version of the MOGQ has excellent reliability (Cronbach α≥.83) and validity [61]. Higher scores reflect stronger motives for online gaming. In this study, the Cronbach α was .95 for the total scale and ranged from 0.84 to 0.89 for each subscale.

#### Depression and Anxiety

The Patient Health Questionnaire-9 (PHQ-9) [62] was used to measure depressive symptoms. It is a diagnostic screening tool that monitors the severity of depression over the last 2 weeks. All items are scored on a 4-point Likert scale (0=Not at all to 3=Nearly every day). The scores range from 0 to 27. Higher scores denote worse depressive symptoms. The Chinese version of the PHQ-9 [63] has suitable reliability (Cronbach α=.85). The Cronbach α was .89 for this study.

The Generalized Anxiety Disorder-7 (GAD-7) [64] was used to measure anxiety symptoms. It is a self-rated scale that assesses the severity of anxiety symptoms over the last 2 weeks. All items are scored on a 4-point Likert scale (0=Not at all to 3=Nearly every day). The scores range from 0 to 21. Higher scores represent worse anxiety symptoms. The Chinese version of the GAD-7 [65] was used, with appropriate internal consistency (Cronbach α=.90) and validity. The Cronbach α was .92 for this study.

### Procedures

The MOGS was translated into Chinese by 2 professional translators using a standard translation and back-translation method [66]. For some controversial items (eg, “Online gaming makes me lose control,” “Online gaming makes my worries more bearable”), we consulted the author of the original scale. Considering the original scale, 2 bilingual psychologists revised the translated version and checked its face validity. A pilot study was conducted with 5 adults and 5 adolescents to test the understandability. Based on their feedback, some descriptions of items were modified, and the final Chinese version of the MOGS (C-MOGS) was created.

We conducted this survey online using Questionnaire Star, a professional online survey platform. By reading recruitment advertisements posted on social networking sites (eg, WeChat, Weibo, and other webcast platforms), individuals could open the questionnaire link. On the first page, participants could read the objectives and content of this research and confirm their participation (minors, those younger than 18 years, had to obtain the consent of their guardian). Each IP address can only be used once to avoid repeated participation. After the questionnaire was submitted, all the data were sent to the researcher's account, and only the researcher could view the data.

### Data Analyses

Data analyses were conducted using SPSS version 25.0 (IBM Corp) and AMOS version 24.0 (IBM Corp). First, basic statistical analyses (eg, descriptive analysis, independent samples *t* tests, chi-square tests) were performed on sociodemographic variables and Internet gaming characteristics. To analyze the construct of the C-MOGS, the total sample was randomly split into 2 subsamples. Sample-1 (n1=390; 229/390, 58.7% male; age: mean 22.25, SD 9.05 years) was used for EFA, and sample-2 (n2=382; 229/382, 59.9% male; age: mean 21.14, SD 8.52 years) was used for CFA. Except for EFA and CFA, all analyses were conducted on data from the entire sample. Independent samples *t* tests and chi-square tests showed that there were no significant differences between the 2 subsamples regarding age (*t*_770_=–1.754, *P*=.08), gender (*χ*^2^_1_=0.121, *P*=.73), average weekly gaming time (*t*_770_=0.775, *P*=.44), gaming devices (χ^2^_1_<1.012, *P*=.31), long-term gaming partner (χ^2^_3_=4.046, *P*=.26), and other demographic variables (*P*=.33-.88). An EFA with principal component analysis (PCA) and varimax-rotation method was conducted on the C-MOGS items. To validate the models derived from the EFA, a CFA was completed with AMOS 24.0 using the maximum likelihood method. The model fit was appraised using multiple fit indexes, including chi-squared:degree of freedom ratio (χ^2^/df<5), goodness-of-fit index (GFI>0.90), Tucker-Lewis Index (TLI>0.90), comparative fit index (CFI>0.90), standardized root of the mean square residual (SRMR<0.08), root mean square error of approximation (<0.05=close fit; <0.08=acceptable fit; <0.1=mediocre fit) [67]. The reliability of the C-MOGS was examined by assessing the internal consistency of the scale and subscale. Acceptable values for the Cronbach α and Guttman split-half coefficients are >0.70, while values >0.80 are considered good [68]. Finally, to test the concurrent and incremental validity, correlation analyses and hierarchical multiple regression analyses were conducted between the C-MOGS and Internet gaming characteristics (gaming time, IGD, gaming motives) as well as anxiety and depression.

## Results

### Sample Characteristics

In this study, analysis was conducted on data from 772 participants (458 men, 59.3%) aged between 13 years and 57 years (age: mean 21.70, SD 8.81 years; participants aged 13-17 years: 281/772, 36.4%). The majority of them were students (555/772, 71.9%). Smartphones were the most popular device for gaming (705/772, 91.3%). Of the sample, 69.4% (536/772) had one or more long-term gaming partners. Participants spent an average of 13.43 (SD 10.88) hours every week playing games. More details on the sample characteristics are shown in Table 1.

**Table 1:** Sociodemographic and Internet gaming characteristics of the sample (n=772).

Characteristic			Participants’ results
**Gender, n (%)**			
	Male	458 (59.3)	
	Female	314 (40.7)	
Age (years), mean (SD)			21.70 (8.81)
**Employment** **, n (%)**			
	Student	555 (71.9)	
	Full-time employee	186 (24.1)	
	Part-time employee	13 (1.7)	
	Unemployed	18 (2.3)	
**Length of education (years), n (%)**			
	≤12	339 (43.9)	
	>12	433 (56.1)	
**Single child, n (%)**			
	Yes	289 (37.4)	
	No	483 (62.6)	
**Gaming devices, n (%)**			
	Smartphone	705 (91.3)	
	Computer	278 (36.1)	
	Tablet	125 (16.2)	
	Game console	51 (6.6)	
**Long-term gaming partners, n (%)**			
	None	236 (30.6)	
	≥1 and <3	198 (25.6)	
	≥3 and <6	159 (20.6)	
	≥6	179 (23.2)	
**Self-evaluation of gaming addiction, n (%)**			
	Yes	129 (16.7)	
	No idea	203 (26.3)	
	No	440 (57.0)	
Weekly gaming time (hours), mean (SD)			13.43 (10.88)

### Factorial Structure of the C-MOGS

#### EFA

To identify the potential factorial structure of the C-MOGS, an EFA was performed on data from sample-1 (n=390). First, we used the Kaiser-Meyer-Olkin (KMO) and Bartlett tests of sphericity to ensure that the sample was suitable for EFA. The KMO value was 0.894, and the Bartlett test of sphericity was significant (χ^2^_66_=3159.742, *P*<.001), confirming the data were sufficient.

The initial analysis extracted 2 factors using the criteria of an eigenvalue>1 and factor loading>0.40. The 2-factor solution (eigenvalues of 5.573 and 2.597) accounted for 68.08% of the total variance, and the loading of all the items was >0.4 (0.646-0.918; Table 2). Factor 1 included items 1 through 6, referred to as the N-MOG; factor 2 included items 7 through 12, which described the P-MOG.

Additionally, according to the dimension of the original scale [13], we also conducted a PCA by setting 3 factors to be extracted. The 3-factor solution (eigenvalues of 5.573, 2.597, and 0.797) explained 74.73% of the total variance (37.44%, 19.31%, and 17.98%, respectively). Item-factor loadings are presented in Table 2. The factors were as follows: factor 1 (items 1, 2, and 3) referred to the N-MOGU; factor 2 (items 4, 5, and 6) was related to the N-MOGD; and factor 3 (items 7, 8, 9, 10, 11, and 12) was related to the P-MOG [54].

**Table 2:** Item-factor loadings of the Chinese version of the Metacognitions about Online Gaming Scale (C-MOGS) based on exploratory factor analyses (sample-1, n=390).

Items	2-factor model			3-factor model			
	F1^a,b^	F2^b,c^	Communality	F1^b,d^	F2^b,e^	F3^b,c^	Communality
(1) I continue to play despite I think it would be better to stop	0.646	0.215	0.463	0.809	0.110	0.178	0.698
(2) I have no control over how much time I play	0.830	0.177	0.721	0.808	0.370	0.151	0.813
(3) Once I start online gaming, I cannot stop	0.808	0.178	0.684	0.743	0.403	0.157	0.739
(4) Online gaming makes me lose control	0.746	0.108	0.569	0.213	0.840	0.134	0.768
(5) Thoughts about online gaming interfere with my functioning	0.743	0.007	0.552	0.222	0.825	0.032	0.731
(6) Thoughts about online gaming are becoming an obsession	0.776	0.165	0.630	0.474	0.624	0.169	0.643
(7) Online gaming makes my worries more bearable	0.275	0.734	0.614	0.190	0.204	0.735	0.618
(8) Online gaming reduces my negative feelings	0.131	0.882	0.796	0.087	0.104	0.885	0.801
(9) Online gaming helps me to control my negative thoughts	0.093	0.918	0.852	0.125	0.014	0.915	0.852
(10) Online gaming stops me from worrying	0.116	0.849	0.734	0.083	0.087	0.850	0.737
(11) Online gaming reduces my anxious feelings	0.107	0.917	0.853	0.081	0.076	0.919	0.856
(12) Online gaming distracts my mind from problems	0.222	0.808	0.703	0.256	0.064	0.800	0.710

^a^Negative Metacognitions about Online Gaming (N-MOG).

^b^Factor loadings present the factor matrix values.

^c^Positive Metacognitions about Online Gaming (P-MOG).

^d^Negative Metacognitions about the Uncontrollability of Online Gaming (N-MOGU).

^e^Negative Metacognitions about the Dangers of Online Gaming (N-MOGD).

#### Confirmatory Factor Analysis

To further evaluate the structural validity of the C-MOGS, we conducted a CFA on sample-2 (n=382) using AMOS 25.0. We compared the goodness of model fit between the 2 aforementioned models. We first tested the 2-factor model, which had a substandard fit in some indexes: χ^2^/df=3.962 and root mean squared error of approximation (RMSEA)=0.880. In comparison, the 3-factor model showed an adequate model fit: χ^2^/df=3.477, GFI=0.929, CFI=0.958, TLI=0.945, SRMR=0.065, RMSEA=0.081 (Table 3). The correlations between P-MOG, N-MOGU, and N-MOGD were moderate (r=0.389 and 0.377, respectively) and were relatively strong between N-MOGU and N-MOGD (r=0.905).

**Figure 1:**
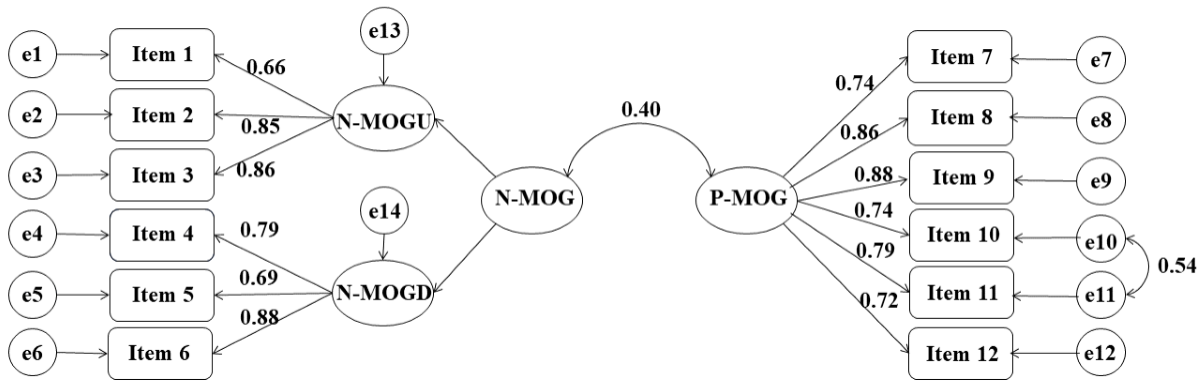
The bifactor model of the Chinese version of the Metacognitions about Online Gaming Scale (C-MOGS), showing the latent factors as ovals, the 12 items in the C-MOGS as rectangles, the error terms (e1-e14) as circles, and the standardized factor loading above the arrows. N-MOG: Negative Metacognitions about Online Gaming; N-MOGD: Negative Metacognitions about the Dangers of Online Gaming; N-MOGU: Negative Metacognitions about the Uncontrollability of Online Gaming; P-MOG: Positive Metacognitions about Online Gaming.

Due to the high correlation between the 2 negative metacognitive factors, we also created a bifactor model (Figure 1), in which N-MOGU and N-MOGD loaded on a second-order factor (N-MOG) and P-MOG was a first-order factor. In this model, the goodness of model fit was the same as that of the 3-factor model (Table 3), and the correlation between N-MOG and P-MOG was moderate (r=0.396).

**Table 3:** Model fit indices of the confirmatory factor analyses for the Chinese version of the Metacognitions about Online Gaming Scale (C-MOGS; Sample 2, n=382).

Model	χ^2^ (df)	χ^2^/df	GFI^a^	CFI^b^	TLI^c^	SRMR^d^	RMSEA^e^
2-factor model	205.517 (52)	3.962	0.917	0.948	0.935	0.067	0.880
3-factor model	173.867 (50)	3.477	0.929	0.958	0.945	0.065	0.810
Bifactor model	173.867 (50)	3.477	0.929	0.958	0.945	0.065	0.810

^a^GFI: goodness-of-fit index.

^b^CFI: comparative fit index.

^c^TLI: Tucker-Lewis Index.

^d^SRMR: standardized root of the mean square residual.

^e^RMSEA: root mean square error of approximation.

### Reliability

The Cronbach α coefficient and split-half reliability were calculated for the scale and its subscales in the total sample (n=772). The α coefficient for the total scale was .894, and it was .823 for the N-MOGU, .799 for the N-MOGD, and .925 for the P-MOG. No item deletion improved the internal consistency. The Guttman split-half coefficient of the overall scale was 0.942, and for each dimension, the coefficients were 0.776, 0.754, and 0.841. These findings confirmed that the C-MOGS and its subscales exhibit adequate internal consistency.

Moreover, we calculated the correlation coefficient between each item and its relative factor scores. The results showed that the item-total correlations for all items were high (r≥0.551).

### Concurrent Validity

We further analyzed the correlation between the 3 factors of the C-MOGS and IGD, gaming motives, anxiety, and depression to test the concurrent validity. Based on the Shapiro-Wilk test, these variables did not follow a normal distribution (all *Ps*<.05). Therefore, Spearman correlation analysis was chosen to explore the relationships between the variables. Table 4 shows the descriptive statistics (median and IQR), and Table 5 shows the correlations between the variables. Each factor of the C-MOGS showed positive correlations with the IGDS9-SF, weekly gaming hours, every dimension of the MOGQ, the PHQ-9, and the GAD-7 (r=0.153 to 0.759, all *Ps*<.01). Moreover, the correlation matrix showed positive correlations between the IGDS9-SF and the other variables (r=0.352 to 0.700, all *Ps*<.01).

**Table 4:** Descriptive statistics for the variables (n=772).

Variables			Median (IQR)		Range
IGDS9-SF^a^			17 (10)		9-45
WGH^b^			9 (11)		1-69
**Motives for Online Gaming Questionnaire (MOGQ)**					
	Social	7 (6)		4-20	
	Escape	7 (5)		4-20	
	Competition	7 (6)		4-20	
	Coping	9 (6)		4-20	
	Skill	7 (6)		4-20	
	Fantasy	6 (5)		4-20	
	Recreation	9 (6)		3-15	
PHQ-9^c^			6 (7)		0-27
GAD-7^d^			4 (6)		0-21
N-MOGU^e^			4 (3)		3-12
N-MOGD^f^			4 (2)		3-12
P-MOG^g^			11 (7)		6-24

^a^IGDS9-SF: Internet Gaming Disorder Scale-Short Form.

^b^WGH: weekly gaming hours (average time).

^c^PHQ-9: Patient Health Questionnaire-9.

^d^GAD-7: Generalized Anxiety Disorder-7.

^e^N-MOGU: Metacognitions about the Uncontrollability of Online Gaming.

^f^N-MOGD: Negative Metacognitions about the Dangers of Online Gaming.

^g^P-MOG: Positive Metacognitions about Online Gaming.

**Table 5:** Spearman correlation coefficients among the variables (n=772).

Variables		IGDS9-SF^a^	WGH^b^	MOGQ^c^								PHQ-9^d^		GAD-7^e^		N-MOGU^f^		N-MOGD^g^		P-MOG^h^
				Social	Escape	Competition	Coping	Skill	Fantasy	Recreation										
**IGDS9-SF**																				
	Correlation	1	0.501	0.352	0.589	0.494	0.542	0.380	0.488	0.416	0.466		0.420		0.700		0.587		0.511	
	*P* value	—^i^	<.001	<.001	<.001	<.001	<.001	<.001	<.001	<.001	<.001		<.001		<.001		<.001		<.001	
**WGH**																				
	Correlation	0.501	1	0.196	0.326	0.288	0.353	0.190	0.290	0.386	0.253		0.193		0.364		0.280		0.312	
	*P* value	<.001	—	<.001	<.001	<.001	<.001	<.001	<.001	<.001	<.001		<.001		<.001		<.001		<.001	
**MOGQ: Social**																				
	Correlation	0.352	0.196	1	0.473	0.521	0.544	0.606	0.523	0.378	0.108		0.118		0.246		0.174		0.398	
	*P* value	<.001	<.001	—	<.001	<.001	<.001	<.001	<.001	<.001	.003		.001		<.001		<.001		<.001	
**MOGQ: Escape**																				
	Correlation	0.589	0.326	0.473	1	0.503	0.786	0.563	0.658	0.458	0.412		0.412		0.435		0.352		0.674	
	*P* value	<.001	<.001	<.001	—	<.001	<.001	<.001	<.001	<.001	<.001		<.001		<.001		<.001		<.001	
**MOGQ: Competition**																				
	Correlation	0.494	0.288	0.521	0.503	1	0.544	0.615	0.552	0.467	0.188		0.196		0.372		0.316		0.412	
	*P* value	<.001	<.001	<.001	<.001	—	<.001	<.001	<.001	<.001	<.001		<.001		<.001		<.001		<.001	
**MOGQ: Coping**																				
	Correlation	0.542	0.353	0.544	0.786	0.544	1	0.684	0.636	0.617	0.284		0.297		0.396		0.245		0.759	
	*P* value	<.001	<.001	<.001	<.001	<.001	—	<.001	<.001	<.001	<.001		<.001		<.001		<.001		<.001	
**MOGQ: Skill**																				
	Correlation	0.380	0.190	0.606	0.563	0.615	0.684	1	0.589	0.403	0.121		0.151		0.249		0.153		0.531	
	*P* value	<.001	<.001	<.001	<.001	<.001	<.001	—	<.001	<.001	.001		<.001		<.001		<.001		<.001	
**MOGQ: Fantasy**																				
	Correlation	0.488	0.290	0.523	0.658	0.552	0.636	0.589	1	0.442	0.289		0.295		0.370		0.322		0.476	
	*P* value	<.001	<.001	<.001	<.001	<.001	<.001	<.001	—	<.001	<.001		<.001		<.001		<.001		<.001	
**MOGQ: Recreation**																				
	Correlation	0.416	0.386	0.378	0.458	0.467	0.617	0.403	0.442	1	0.234		0.226		0.348		0.157		0.430	
	*P* value	<.001	<.001	<.001	<.001	<.001	<.001	<.001	<.001	—	<.001		<.001		<.001		<.001		<.001	
**PHQ-9**																				
	Correlation	0.466	0.253	0.108	0.412	0.188	0.284	0.121	0.289	0.234	1		0.789		0.411		0.411		0.270	
	*P* value	<.001	<.001	.003	<.001	<.001	<.001	.001	<.001	<.001	—		<.001		<.001		<.001		<.001	
**GAD-7**																				
	Correlation	0.420	0.193	0.118	0.412	0.196	0.297	0.151	0.295	0.226	0.789		1		0.383		0.376		0.280	
	*P* value	<.001	<.001	.001	<.001	<.001	<.001	<.001	<.001	<.001	<.001		—		<.001		<.001		<.001	
**N-MOGU**																				
	Correlation	0.700	0.364	0.246	0.435	0.372	0.396	0.249	0.370	0.348	0.411		0.383		1		0.530		0.359	
	*P* value	<.001	<.001	<.001	<.001	<.001	<.001	<.001	<.001	<.001	<.001		<.001		—		<.001		<.001	
**N-MOGD**																				
	Correlation	0.587	0.280	0.174	0.352	0.316	0.245	0.153	0.322	0.157	0.411		0.376		0.530		1		0.257	
	*P* value	<.001	<.001	<.001	<.001	<.001	<.001	<.001	<.001	<.001	<.001		<.001		<.001		—		<.001	
**P-MOG**																				
	Correlation	0.511	0.312	0.398	0.674	0.412	0.759	0.531	0.476	0.430	0.270		0.280		0.359		0.257		1	
	*P* value	<.001	<.001	<.001	<.001	<.001	<.001	<.001	<.001	<.001	<.001		<.001		<.001		<.001		—	

^a^IGDS9-SF: Internet Gaming Disorder Scale-Short Form.

^b^WGH: weekly gaming hours (average time).

^c^Motives for Online Gaming Questionnaire.

^d^PHQ-9: Patient Health Questionnaire-9.

^e^GAD-7: Generalized Anxiety Disorder-7.

^f^N-MOGU: Metacognitions about the Uncontrollability of Online Gaming.

^g^N-MOGD: Negative Metacognitions about the Dangers of Online Gaming.

^h^P-MOG: Positive Metacognitions about Online Gaming.

^i^Not applicable.

### Incremental Validity

We conducted a hierarchical multiple linear regression analysis to identify the incremental effect of metacognitions about online gaming on IGD. The IGDS9-SF was the outcome variable, and the 3 factors of the C-MOGS were predictor variables, along with other variables related to the IGDS9-SF (gender, weekly gaming hours, the 7 factors of the MOGQ, and the total PHQ-9 and GAD-7 scores). Each variable was input in the following order: step 1: age and gender (0=female, 1=male); step 2: weekly gaming hours; step 3: the 7 factors of the MOGQ; step 4: GAD-7, PHQ-9; step 5: the 3 dimensions of the C-MOGS.

The Durbin-Watson statistic showed that the observed values were independent of each other (D-W=2.077). All tolerance values were above 0.1 (0.180-0.878), indicating no multicollinearity. The results are presented in Table 6. The 3 factors of the C-MOGS accounted for 13.0% of the variance in the IGDS9-SF (*P*<.001). In step 5, the final model indicated that gender, weekly gaming hours, the PHQ-9 score, the MOGQ-Escape score, the MOGQ-Competition score, and the factors of the C-MOGS were significant positive predictors of the IGDS9-SF (R^2^=0.729, *P*<.001, adjusted R^2^=0.724), and the most important predictor was the N-MOGU (β=0.326, *P*<.001).

**Table 6:** Hierarchical multiple regression analyses with the Internet Gaming Disorder Scale-Short Form (IGDS9-SF) as the outcome variable and the Chinese version of the Metacognitions about Online Gaming Scale (C-MOGS) factors as predictor variables, together with gender, weekly gaming hours, motives related to online gaming, depression, and anxiety (n=772).

Variable		Step 1^a^				Step 2^b^				Step 3^c^				Step 4^d^				Step 5^e^		
		β	T	*P* value	β		T	*P* value	β		T	*P* value	β		T	*P* value	β		T	*P* value
Age		–0.056	–1.551	.12	–0.110		–3.633	<.001	–0.068		–2.640	.008	–0.050		–2.044	.04	0.004		0.206	.84
Gender		0.204	5.694	<.001	0.110		3.603	<.001	0.084		3.170	.002	0.114		4.588	<.001	0.076		3.712	<.001
WGH^f^		—^g^	—	—	0.540		17.971	<.001	0.315		11.137	<.001	0.277		10.309	<.001	0.155		6.705	<.001
**Motives for Online Gaming Questionnaire**																				
	Social	—	—	—	—		—	—	–0.019		–0.567	.57	0.008		0.241	.81	0.010		0.387	.70
	Escape	—	—	—	—		—	—	0.381		8.805	<.001	0.265		6.328	<.001	0.132		3.743	<.001
	Competition	—	—	—	—		—	—	0.163		4.740	<.001	0.149		4.632	<.001	0.066		2.458	.01
	Coping	—	—	—	—		—	—	0.022		0.425	.67	0.016		0.333	.74	–0.022		–0.493	.62
	Skill	—	—	—	—		—	—	–0.106		–2.607	.009	–0.058		–1.529	.13	0.024		0.761	.45
	Fantasy	—	—	—	—		—	—	0.098		2.601	.009	0.068		1.916	.056	0.020		0.688	.49
	Recreation	—	—	—	—		—	—	0.017		0.515	.61	0.009		0.299	.77	0.016		0.618	.54
PHQ-9^h^		—	—	—	—		—	—	—		—	—	0.224		5.553	<.001	0.104		3.091	.002
GAD-7^i^		—	—	—	—		—	—	—		—	—	0.073		1.847	.07	0.025		0.784	.43
N-MOGU^j^		—	—	—	—		—	—	—		—	—	—		—	—	0.326		11.286	<.001
N-MOGD^k^		—	—	—	—		—	—	—		—	—	—		—	—	0.206		7.248	<.001
P-MOG^l^		—	—	—	—		—	—	—		—	—	—		—	—	0.089		2.969	.003

^a^R^2^=0.049; adjusted R^2^=0.047; ΔR^2^=0.049; *P*<.001.

^b^R^2^=0.331; adjusted R^2^=0.328; ΔR^2^=0.281; *P*<.001.

^c^R^2^=0.539; adjusted R^2^=0.533; ΔR^2^=0.208; *P*<.001.

^d^R^2^=0.599; adjusted R^2^=0.593; ΔR^2^=0.061; *P*<.001.

^e^R^2^=0.729; adjusted R^2^=0.724; ΔR^2^=0.130; *P*<.001.

^f^WGH: weekly gaming hours (average time).

^g^Not applicable.

^h^PHQ-9: Patient Health Questionnaire-9.

^i^GAD-7: Generalized Anxiety Disorder-7.

^j^N-MOGU: Metacognitions about the Uncontrollability of Online Gaming.

^k^N-MOGD: Negative Metacognitions about the Dangers of Online Gaming.

^l^P-MOG: Positive Metacognitions about Online Gaming.

## Discussion

### Principal Findings

To investigate the psychometric properties of the Chinese MOGS and its association with IGD, this study translated and tested it in China for the first time. In general, the results suggested that the C-MOGS could potentially serve as a valid and reliable tool to assess specific metacognitions about online gaming and it may have the capacity to predict IGD independently.

First, factor analyses were used to explore the structural validity of the scale. The EFA suggested a 2-factor solution (N-MOG and P-MOG), which was consistent with the first assumption of the original scale [13]. By setting 3 factors to be extracted, the EFA also obtained the same 3-factor solution as the final version of the original scale (N-MOGU, N-MOGD, and P-MOG) [13,54]. Through CFA, the 3-factor model was later proved to have the best data fit. Moreover, we attempted to build a bifactor model that included a first-order factor (P-MOG) and a second-order factor (N-MOG: N-MOGU and N-MOGD). This model had the same goodness of model fit as the 3-factor structure. To maintain consistency with the original scale, the 3-factor structure is recommended for measuring specific online gaming metacognitions in the Chinese population. For studies that compare N-MOG and P-MOG, the bifactor model can be considered.

The 3-factor structure of the C-MOGS demonstrated adequate internal consistency, with Cronbach α coefficients ranging from .799 to .925 for each factor and the full scale, along with Guttman split-half coefficients ranging from 0.754 to 0.942. The current findings also provide evidence for the concurrent and incremental validity of the C-MOGS. Each subscale was significantly positively correlated with IGD, weekly gaming hours, gaming motives, depression, and anxiety. Moreover, the C-MOGS accounted for 13.0% of the variance in IGD while controlling for other variables. These findings highlight the utility of the C-MOGS as a reliable and valid tool to assess metacognitions about online gaming among the Chinese population.

Furthermore, this study explored the effects of metacognitions about online gaming, gaming motives, anxiety, and depression on IGD using hierarchical multiple linear regression analysis. After adding metacognitions about online gaming to the regression equations, the final model accounted for 72.9% of the variance in IGD. In addition to metacognitions, gender, weekly gaming hours, escapism motives, competition motives, and depression significantly predicted IGD, suggesting that these factors collectively contribute to the development and maintenance of IGD [69-71]. Importantly, the inclusion of metacognitions led to a reduction in the standardized regression coefficients of these variables, and the predictive effect of anxiety on IGD became nonsignificant. This indicates that metacognition may partially mediate or explain the impact of these factors on IGD. This finding is consistent with previous research, suggesting that metacognitions about online gaming may mediate the influence of other psychological factors, such as psychological dependence, anxiety, and depression, on IGD [55,72-75]. These results indicate that specific metacognitions about online gaming are important predictors of IGD, which is consistent with previous studies [13,54,55]. However, the mechanisms underlying the role of metacognitions in IGD seem to be interrelated with other psychological factors, which remains inconclusive.

In a hypothesized model, metacognitions about online gaming may promote problematic gaming engagement by increasing gaming time and disrupting normal emotion and cognition [76]. Consistent with this view, our study found that people with more metacognitions about online gaming would spend more time playing games and feel more anxious and depressed. P-MOG increases gaming time by promoting online gaming as a self-regulation method for emotion and cognition [13,21]. N-MOGU will maintain problematic gaming engagement by destroying one’s confidence in self-control, while N-MOGD can induce negative reinforcement and compulsive gaming engagement by triggering negative emotions such as anxiety and depression [21,76]. Furthermore, gaming motives may be an intermediate factor, as our study found: Gaming motives were simultaneously significantly correlated with MOGS and IGD. Dysfunctional metacognition activates maladaptive coping strategies and motivation, which causes negative emotions to persist and eventually leads to IGD [77]. Moreover, other studies have different views. For example, metacognitions have a mediating effect on the association between emotional dysregulation and problematic Internet use [78], and online gaming thought suppression and impulsiveness mediate the relationship between metacognition and IGD [79]. Therefore, the association between metacognition and IGD cannot be summarized by simple causality. Other psychological variables, such as motives, coping style, impulsiveness, and emotional regulation, should be considered in future research.

Since maladaptive metacognitions are an important predictor of IGD, interventions specifically addressing maladaptive metacognitions, such as metacognition therapy (MCT), may be beneficial for the prevention and treatment of IGD. MCT, an intervention aimed at modifying dysfunctional metacognition, is effective for treating psychiatric and psychological diseases such as anxiety, depression, and schizophrenia [80-84]. Although MCT is not widely used in the treatment of addictive behaviors, researchers are attempting to prove its efficacy [11]. In some pilot studies, MCT was used to effectively treat alcohol abuse and gambling disorder [85,86]. However, the specific efficacy of MCT for treating IGD needs to be further verified in clinical research. This study provides evidence for the potential value of MCT in the clinical treatment of IGD and offers an effective tool for conducting MCT for IGD specifically in the Chinese population.

### Limitations

Although this study has the advantages of a large sample size with people of different ages, it has several limitations that should be considered. First, this study adopted convenience sampling instead of random sampling, and only gamers were included. Therefore, it does not sufficiently represent all Chinese people. Second, collecting data using an online self-report questionnaire may increase the probability of participants giving false answers. However, this procedure is reported to be as reliable as pencil-and-paper surveys [87], which is likely to reduce social desirability and increase levels of honesty [88]. Third, this study lacked test-retest reliability of the C-MOGS; further research is required to test its stability. Finally, as a cross-sectional study, we could not infer the causality of the studied variables. Thus, longitudinal research is needed to further explore the relationship between metacognition and IGD.

### Conclusion

In summary, this study offers some evidence that supports the satisfactory psychometric properties of the C-MOGS and highlights the possibility of metacognition as an independent risk factor in gaming behavior. It may be a useful and prospective tool for exploring psychological mechanisms of IGD and helping health professionals identify risky gamers (eg, individuals with more metacognitions about online gaming, specifically negative metacognitions about the uncontrollability of online gaming). Additionally, MCT may be beneficial for the prevention and treatment of IGD. This study may support more attention for metacognitive beliefs in addictive behaviors.

## Abbreviations

CAS: cognitive attentional syndrome
CFA: confirmatory factor analysis
CFI: comparative fit index
C-MOGS: Chinese version of the Metacognitions about Online Gaming Scale
EFA: exploratory factor analysis
GAD-7: Generalized Anxiety Disorder-7
GFI: goodness-of-fit index
*ICD-11*: *Eleventh Revision of the International Classification of Diseases*
IGD: Internet Gaming Disorder
IGDS9-SF: Internet Gaming Disorder Scale-Short Form
I-PACE: Interaction of Person-Affect-Cognition-Execution
KMO: Kaiser-Meyer-Olkin
MCT: metacognition therapy
MOGQ: Motives for Online Gaming Questionnaire
MOGS: Metacognitions about Online Gaming Scale
N-MOG: Negative Metacognitions about Online Gaming
N-MOGD: Negative Metacognitions about the Dangers of Online Gaming
N-MOGU: Negative Metacognitions about the Uncontrollability of Online Gaming
PCA: principal component analysis
PHQ-9: Patient Health Questionnaire-9
P-MOG: Positive Metacognitions about Online Gaming
RMSEA: root mean squared error of approximation
SRMR: standardized root of the mean square residual
TLI: Tucker-Lewis Index

## References

[1] Flavell JH (1979). Metacognition and cognitive monitoring: A new area of cognitive-developmental inquiry. American Psychologist.

[2] Metcalfe J (2009). Metacognitive judgments and control of study. Curr Dir Psychol Sci.

[3] Fernandez-Duque D, Baird JA, Posner MI (2000). Executive attention and metacognitive regulation. Conscious Cogn.

[4] Heyes C, Bang D, Shea N, Frith CD, Fleming SM (2020). Knowing ourselves together: the cultural origins of metacognition. Trends Cogn Sci.

[5] Schraw G, Moshman D (1995). Metacognitive theories. Educ Psychol Rev.

[6] Rabbitt PM (1966). Error correction time without external error signals. Nature.

[7] Bryce D, Whitebread D, Szűcs D (2014). The relationships among executive functions, metacognitive skills and educational achievement in 5 and 7 year-old children. Metacognition Learning.

[8] Adam KCS, Vogel EK (2017). Confident failures: Lapses of working memory reveal a metacognitive blind spot. Atten Percept Psychophys.

[9] Wells A, Matthews G (1994). Attention and Emotion: A Clinical Perspective.

[10] Myers EJ, Abel DB, Mickens JL, Russell MT, Rand KL, Salyers MP, Lysaker PH, Minor KS (2024). Meta-analysis of the relationship between metacognition and disorganized symptoms in psychosis. Schizophr Res.

[11] Hamonniere T, Varescon I (2018). Metacognitive beliefs in addictive behaviours: A systematic review. Addict Behav.

[12] Casale S, Musicò A, Spada MM (2021). A systematic review of metacognitions in Internet Gaming Disorder and problematic Internet, smartphone and social networking sites use. Clin Psychol Psychother.

[13] Spada MM, Caselli G (2017). The Metacognitions about Online Gaming Scale: Development and psychometric properties. Addict Behav.

[14] Wells A, Matthews G (1996). Modelling cognition in emotional disorder: the S-REF model. Behav Res Ther.

[15] Wells A (2002). Emotional disorders and metacognition: innovative cognitive therapy. Psychiatric Ment Health Nurs.

[16] Wells A (2002). The Self-Regulatory Executive Function (S-REF) Model. Emotional Disorders and Metacognition: Innovative Cognitive Therapy.

[17] Sun X, Zhu C, So SHW (2017). Dysfunctional metacognition across psychopathologies: A meta-analytic review. Eur Psychiatry.

[18] Hoven M, Lebreton M, Engelmann JB, Denys D, Luigjes J, van Holst RJ (2019). Abnormalities of confidence in psychiatry: an overview and future perspectives. Transl Psychiatry.

[19] Rosello R, Martinez-Raga J, Tomas JM, Mira A, Cortese S (2023). Cognitive and behavioral profiles in children with autism spectrum disorder with and without attention-deficit/hyperactivity disorder. Child Adolesc Ment Health.

[20] Lungu PF, Lungu C, Ciobîcă A, Balmus IM, Boloș A, Dobrin R, Luca AC (2023). Metacognition in schizophrenia spectrum disorders-current methods and approaches. Brain Sci.

[21] Spada MM, Caselli G, Nikčević AV, Wells A (2015). Metacognition in addictive behaviors. Addict Behav.

[22] Spada MM, Wells A (2006). Metacognitions about alcohol use in problem drinkers. Clin Psychology and Psychoth.

[23] Lindberg A, Fernie BA, Spada MM (2011). Metacognitions in problem gambling. J Gambl Stud.

[24] Gierski F, Spada MM, Fois E, Picard A, Naassila M, Van der Linden M (2015). Positive and negative metacognitions about alcohol use among university students: Psychometric properties of the PAMS and NAMS French versions. Drug Alcohol Depend.

[25] Dragan WL, Domozych W, Czerski PM, Dragan M (2018). Positive metacognitions about alcohol mediate the relationship between variability and problematic drinking in a sample of young women. Neuropsychiatr Dis Treat.

[26] Khosravani V, Zandifar A, Sharifi Bastan F, Kolubinski DC, Amirinezhad A (2020). Psychometric properties of the Persian versions of the Positive Alcohol Metacognitions Scale (Persian-PAMS) and the Negative Alcohol Metacognitions Scale (Persian-NAMS) in alcohol-dependent individuals. Addict Behav.

[27] Laghi F, Pompili S, Bianchi D, Lonigro A, Baiocco R (2020). Dysfunctional metacognition processes as risk factors for drunkorexia during adolescence. J Addict Dis.

[28] Alma L, Spada MM, Fernie BA, Yilmaz-Samanci AE, Caselli G, Nikčević AV (2018). Metacognitions in smoking: Evidence from a cross-cultural validation of the metacognitions about smoking questionnaire in a Turkish sample. Psychiatry Res.

[29] Nikčević AV, Alma L, Marino C, Kolubinski D, Yılmaz-Samancı AE, Caselli G, Spada MM (2017). Modelling the contribution of negative affect, outcome expectancies and metacognitions to cigarette use and nicotine dependence. Addict Behav.

[30] Caselli G, Fernie B, Canfora F, Mascolo C, Ferrari A, Antonioni M, Giustina L, Donato G, Marcotriggiani A, Bertani A, Altieri A, Pellegrini E, Spada MM (2018). The Metacognitions about Gambling Questionnaire: Development and psychometric properties. Psychiatry Res.

[31] Rogier G, Beomonte Zobel S, Morganti W, Ponzoni S, Velotti P (2021). Metacognition in gambling disorder: A systematic review and meta-analysis. Addict Behav.

[32] Hoven M, de Boer NS, Goudriaan AE, Denys D, Lebreton M, van Holst RJ, Luigjes J (2022). Metacognition and the effect of incentive motivation in two compulsive disorders: Gambling disorder and obsessive-compulsive disorder. Psychiatry Clin Neurosci.

[33] Hamidi F, Ghasedi J (2020). Cognitive and metacognitive impairments of drug addicted, internet addicted and normal individuals in youth ages: a comparative study. Int J High Risk Behav Addict.

[34] Seyed Hashemi SG, Hosseinnezhad S, Dini S, Griffiths MD, Lin C, Pakpour AH (2020). The mediating effect of the cyberchondria and anxiety sensitivity in the association between problematic internet use, metacognition beliefs, and fear of COVID-19 among Iranian online population. Heliyon.

[35] Akbari M, Bahadori MH, Mohammadkhani S, Kolubinski DC, Nikčević AV, Spada MM (2021). A discriminant analysis model of psychosocial predictors of problematic Internet use and cannabis use disorder in university students. Addict Behav Rep.

[36] Balıkçı K, Aydın O, Sönmez İ, Kalo B, Ünal-Aydın P (2020). The relationship between dysfunctional metacognitive beliefs and problematic social networking sites use. Scand J Psychol.

[37] Casale S, Caponi L, Fioravanti G (2020). Metacognitions about problematic smartphone use: Development of a self-report measure. Addict Behav.

[38] Ünal-Aydın P, Obuća F, Aydın O, Spada MM (2021). The role of metacognitions and emotion recognition in problematic SNS use among adolescents. J Affect Disord.

[39] Aydın O, Güçlü M, Ünal-Aydın P, Spada MM (2020). Metacognitions and emotion recognition in Internet Gaming Disorder among adolescents. Addict Behav Rep.

[40] Zhang MX, Lei LSM, Wells A, Dang L, Wu AMS (2020). Validation of a Chinese version of the short form of Metacognitions Questionnaire (MCQ-30). J Affect Disord.

[41] Petry NM, Rehbein F, Gentile DA, Lemmens JS, Rumpf H, Mößle T, Bischof G, Tao R, Fung DSS, Borges G, Auriacombe M, González Ibáñez A, Tam P, O'Brien CP (2014). An international consensus for assessing internet gaming disorder using the new DSM-5 approach. Addiction.

[42] Rumpf H, Achab S, Billieux J, Bowden-Jones H, Carragher N, Demetrovics Z, Higuchi S, King DL, Mann K, Potenza M, Saunders JB, Abbott M, Ambekar A, Aricak OT, Assanangkornchai S, Bahar N, Borges G, Brand M, Chan EM, Chung T, Derevensky J, Kashef AE, Farrell M, Fineberg NA, Gandin C, Gentile DA, Griffiths MD, Goudriaan AE, Grall-Bronnec M, Hao W, Hodgins DC, Ip P, Király O, Lee HK, Kuss D, Lemmens JS, Long J, Lopez-Fernandez O, Mihara S, Petry NM, Pontes HM, Rahimi-Movaghar A, Rehbein F, Rehm J, Scafato E, Sharma M, Spritzer D, Stein DJ, Tam P, Weinstein A, Wittchen H, Wölfling K, Zullino D, Poznyak V (2018). Including gaming disorder in the ICD-11: The need to do so from a clinical and public health perspective. J Behav Addict.

[43] Kuss D (2013). Internet gaming addiction: current perspectives. PRBM.

[44] Zheng H, Hu Y, Wang Z, Wang M, Du X, Dong G (2019). Meta-analyses of the functional neural alterations in subjects with Internet gaming disorder: Similarities and differences across different paradigms. Prog Neuropsychopharmacol Biol Psychiatry.

[45] Deng X, Hu Y, Liu C, Li Q, Yang N, Zhang Q, Liu L, Qiu J, Xu H, Xue L, Shi Y, Wang X, Zhao H (2024). Psychological distress and aggression among adolescents with internet gaming disorder symptoms. Psychiatry Res.

[46] Ahmed GK, Abdalla AA, Mohamed AM, Mohamed LA, Shamaa HA (2022). Relation between internet gaming addiction and comorbid psychiatric disorders and emotion avoidance among adolescents: A cross-sectional study. Psychiatry Res.

[47] Stevens MW, Dorstyn D, Delfabbro PH, King DL (2021). Global prevalence of gaming disorder: A systematic review and meta-analysis. Aust N Z J Psychiatry.

[48] Mak K, Lai C, Watanabe H, Kim D, Bahar N, Ramos M, Young KS, Ho RCM, Aum N, Cheng C (2014). Epidemiology of internet behaviors and addiction among adolescents in six Asian countries. Cyberpsychol Behav Soc Netw.

[49] Xiang Y, Jin Y, Zhang L, Li L, Ungvari GS, Ng CH, Zhao M, Hao W (2020). An overview of the expert consensus on the prevention and treatment of gaming disorder in China (2019 edition). Neurosci Bull.

[50] Liao Z, Huang Q, Huang S, Tan L, Shao T, Fang T, Chen X, Lin S, Qi J, Cai Y, Shen H (2020). Prevalence of internet gaming disorder and its association with personality traits and gaming characteristics among Chinese adolescent gamers. Front Psychiatry.

[51] Ji Y, Yin MXC, Zhang AY, Wong DFK (2022). Risk and protective factors of Internet gaming disorder among Chinese people: A meta-analysis. Aust N Z J Psychiatry.

[52] Brand M, Young KS, Laier C, Wölfling K, Potenza MN (2016). Integrating psychological and neurobiological considerations regarding the development and maintenance of specific Internet-use disorders: An Interaction of Person-Affect-Cognition-Execution (I-PACE) model. Neurosci Biobehav Rev.

[53] Brand M, Wegmann E, Stark R, Müller A, Wölfling K, Robbins TW, Potenza MN (2019). The Interaction of Person-Affect-Cognition-Execution (I-PACE) model for addictive behaviors: Update, generalization to addictive behaviors beyond internet-use disorders, and specification of the process character of addictive behaviors. Neurosci Biobehav Rev.

[54] Akbari M, Bahadori MH, Bouruki Milan B, Caselli G, Spada MM (2021). Metacognitions as a predictor of online gaming in adolescents: Psychometric properties of the metacognitions about online gaming scale among Iranian adolescents. Addict Behav.

[55] Marino C, Canale N, Vieno A, Caselli G, Scacchi L, Spada MM (2020). Social anxiety and Internet gaming disorder: The role of motives and metacognitions. J Behav Addict.

[56] Pontes HM, Griffiths MD (2015). Measuring DSM-5 internet gaming disorder: Development and validation of a short psychometric scale. Computers in Human Behavior.

[57] Poon LYJ, Tsang HWH, Chan TYJ, Man SWT, Ng LY, Wong YLE, Lin C, Chien C, Griffiths MD, Pontes HM, Pakpour AH (2021). Psychometric properties of the Internet Gaming Disorder Scale-Short-Form (IGDS9-SF): systematic review. J Med Internet Res.

[58] Yam C, Pakpour AH, Griffiths MD, Yau W, Lo CM, Ng JMT, Lin C, Leung H (2019). Psychometric testing of three Chinese online-related addictive behavior instruments among Hong Kong university students. Psychiatr Q.

[59] Qin L, Cheng L, Hu M, Liu Q, Tong J, Hao W, Luo T, Liao Y (2020). Clarification of the cut-off score for nine-item Internet Gaming Disorder Scale-Short Form (IGDS9-SF) in a Chinese context. Front Psychiatry.

[60] Demetrovics Z, Urbán R, Nagygyörgy K, Farkas J, Zilahy D, Mervó B, Reindl A, Ágoston C, Kertész A, Harmath E (2011). Why do you play? The development of the motives for online gaming questionnaire (MOGQ). Behav Res Methods.

[61] Wu AMS, Lai MHC, Yu S, Lau JTF, Lei M (2017). Motives for online gaming questionnaire: Its psychometric properties and correlation with Internet gaming disorder symptoms among Chinese people. J Behav Addict.

[62] Kroenke K, Spitzer RL, Williams JB (2001). The PHQ-9: validity of a brief depression severity measure. J Gen Intern Med.

[63] Zhang Y, Liang W, Chen Z, Zhang H, Zhang J, Weng X, Yang S, Zhang L, Shen L, Zhang Y (2013). Validity and reliability of Patient Health Questionnaire-9 and Patient Health Questionnaire-2 to screen for depression among college students in China. Asia Pac Psychiatry.

[64] Spitzer RL, Kroenke K, Williams JBW, Löwe B (2006). A brief measure for assessing generalized anxiety disorder: the GAD-7. Arch Intern Med.

[65] Zhou Y, Xu J, Rief W (2020). Are comparisons of mental disorders between Chinese and German students possible? An examination of measurement invariance for the PHQ-15, PHQ-9 and GAD-7. BMC Psychiatry.

[66] Beaton DE, Bombardier C, Guillemin F, Ferraz MB (2000). Guidelines for the process of cross-cultural adaptation of self-report measures. Spine (Phila Pa 1976).

[67] Hu L, Bentler PM (1999). Cutoff criteria for fit indexes in covariance structure analysis: Conventional criteria versus new alternatives. Structural Equation Modeling: A Multidisciplinary Journal.

[68] Cortina JM (1993). What is coefficient alpha? An examination of theory and applications. Journal of Applied Psychology.

[69] Li Y, Tang Y, Huang S, Tan L, Huang Q, Chen X, Lin S, Hao J, Liao Z, Shen H (2023). Role of gaming devices associated with Internet gaming disorder in China: cross-sectional study. JMIR Serious Games.

[70] Sit HF, Chang CI, Yuan GF, Chen C, Cui L, Elhai JD, Hall BJ (2023). Symptoms of internet gaming disorder and depression in Chinese adolescents: A network analysis. Psychiatry Res.

[71] Wang H, Cheng C (2022). The associations between gaming motivation and internet gaming disorder: systematic review and meta-analysis. JMIR Ment Health.

[72] Dang L, Yang HM, Spada MM, Wu AMS (2024). A three-wave longitudinal study on the underlying metacognitive mechanism between depression and Internet gaming disorder. J Behav Addict.

[73] Efrati Y, Spada MM (2023). "I have no control over how much time I play" the metacognitions about online gaming scale: Evidence from a cross-cultural validation among Israeli adolescents. Addict Behav.

[74] Lin S, Tan L, Chen X, Liao Z, Li Y, Tang Y, Shi Y, Hao J, Wang X, Huang Q, Shen H (2023). Emotion dysregulation and Internet gaming disorder in young people: Mediating effects of negative affect and metacognitions. J Affect Disord.

[75] Gandolfi E, Soyturk I, Ferdig RE (2021). Evaluating U.S. gamers' metacognitions about digital entertainment: Validation of Metacognition about Online Gaming Scale in the U.S. context. J Affect Disord.

[76] Caselli G, Marino C, Spada MM (2020). Modelling online gaming metacognitions: the role of time spent gaming in predicting problematic Internet use. J Rat-Emo Cognitive-Behav Ther.

[77] Marino C, Spada MM (2017). Dysfunctional cognitions in online gaming and Internet gaming disorder: a narrative review and new classification. Curr Addict Rep.

[78] Akbari M (2017). Metacognitions or distress intolerance: The mediating role in the relationship between emotional dysregulation and problematic internet use. Addict Behav Rep.

[79] Efrati Y, Kolubinski DC, Marino C, Spada MM (2021). Modelling the contribution of metacognitions, impulsiveness, and thought suppression to behavioural addictions in adolescents. Int J Environ Res Public Health.

[80] Balzan RP, Mattiske JK, Delfabbro P, Liu D, Galletly C (2019). Individualized metacognitive training (MCT+) reduces delusional symptoms in psychosis: a randomized clinical trial. Schizophr Bull.

[81] McEvoy PM (2019). Metacognitive therapy for anxiety disorders: a review of recent advances and future research directions. Curr Psychiatry Rep.

[82] Jelinek L, Van Quaquebeke N, Moritz S (2017). Cognitive and metacognitive mechanisms of change in metacognitive training for depression. Sci Rep.

[83] Cheli S, Cavalletti V, Lysaker PH, Dimaggio G, Petrocchi N, Chiarello F, Enzo C, Velicogna F, Mancini F, Goldzweig G (2023). A pilot randomized controlled trial comparing a novel compassion and metacognition approach for schizotypal personality disorder with a combination of cognitive therapy and psychopharmacological treatment. BMC Psychiatry.

[84] Hotte-Meunier A, Penney D, Mendelson D, Thibaudeau E, Moritz S, Lepage M, Sauvé G (2023). Effects of metacognitive training (MCT) on social cognition for schizophrenia spectrum and related psychotic disorders: a systematic review and meta-analysis. Psychol. Med.

[85] Caselli G, Martino F, Spada MM, Wells A (2018). Metacognitive therapy for alcohol use disorder: a systematic case series. Front Psychol.

[86] Gehlenborg J, Bücker L, Berthold M, Miegel F, Moritz S (2021). Feasibility, acceptance, and safety of metacognitive training for problem and pathological gamblers (Gambling-MCT): a pilot study. J Gambl Stud.

[87] Weigold A, Weigold IK, Russell EJ (2013). Examination of the equivalence of self-report survey-based paper-and-pencil and internet data collection methods. Psychol Methods.

[88] Wood RTA, Griffiths MD, Eatough V (2004). Online data collection from video game players: methodological issues. Cyberpsychol Behav.

